# STRATEGIES AND TECHNOLOGIES TO SUSTAIN NATIONWIDE IMPLEMENTATION OF ALIVIADO DEMENTIA CARE

**DOI:** 10.1093/geroni/igac059.340

**Published:** 2022-12-20

**Authors:** Abraham Brody, Aditi Durga, Ariel Ford, Rebecca Lassell, Shih-Yin Lin

**Affiliations:** HIGN at the NYU Rory Meyers College of Nursing, New York, New York, United States; New York University, New York, New York, United States; New York University Rory Meyers College of Nursing, New York, New York, United States; New York University, New York, New York, United States; New York University, New York, New York, United States

## Abstract

It is well documented that knowledge improvement alone is not sufficient to sustain systems level changes. Through supporting dementia care workforce training and the embedding of Aliviado program tools (treatment algorithms, assessments, care plans, and caregiver education materials) into hospices’ clinical workflow across the U.S., we learned that both technologies and human support strategies are essential for lasting changes. In this presentation, we first discuss our experience leveraging a powerful customer relationship management software (Salesforce) to assign, manage, and encourage completion of the interprofessional Aliviado Dementia Care training for thousands of active users across the nation. We then share how we provide additional human support and implementation tools in the face of COVID-19, which can inform implementation of similar trials, including office hours, training reports, Plan-Do-Study-Act worksheets, symptom management cheat sheets, champion calls, and electronic health records integration meetings with each agency to locally tailor and embed Aliviado tools.

